# Fine Mapping of a New Major QTL-*qGLS8* for Gray Leaf Spot Resistance in Maize

**DOI:** 10.3389/fpls.2021.743869

**Published:** 2021-09-17

**Authors:** Hongbo Qiu, Chunhong Li, Wenzhu Yang, Kang Tan, Qiang Yi, Mei Yang, Guangxiao Bai

**Affiliations:** College of Agriculture, Guizhou University, Guiyang, China

**Keywords:** maize resistance, gray leaf spot, quantitative trait locus, fine mapping, SSR

## Abstract

Gray leaf spot (GLS), caused by different species of *Cercospora*, is a fungal, non-soil-borne disease that causes serious reductions in maize yield worldwide. The identification of major quantitative trait loci (QTLs) for GLS resistance in maize is essential for developing marker-assisted selection strategies in maize breeding. Previous research found a significant difference (*P* < 0.01) in GLS resistance between T32 (highly resistant) and J51 (highly susceptible) genotypes of maize. Initial QTL analysis was conducted in an F_2 : 3_ population of 189 individuals utilizing genetic maps that were constructed using 181 simple sequence repeat (SSR) markers. One QTL (*qGLS8*) was detected, defined by the markers umc1130 and umc2354 in three environments. The *qGLS8* QTL detected in the initial analysis was located in a 51.96-Mb genomic region of chromosome 8 and explained 7.89–14.71% of the phenotypic variation in GLS resistance in different environments. We also developed a near isogenic line (NIL) BC_3_F_2_ population with 1,468 individuals and a BC_3_F_2_-Micro population with 180 individuals for fine mapping. High-resolution genetic and physical maps were constructed using six newly developed SSRs. The QTL-*qGLS8* was narrowed down to a 124-kb region flanked by the markers ym20 and ym51 and explained up to 17.46% of the phenotypic variation in GLS resistance. The QTL-*qGLS8* contained seven candidate genes, such as an *MYB-related transcription factor 24* and a *C*_3_*H transcription factor 347*), and long intergenic non-coding RNAs (lincRNAs). The present study aimed to provide a foundation for the identification of candidate genes for GLS resistance in maize.

## Key Notes

A new major quantitative trait locus for resistance to maize gray leaf spot (QTL-*qGLS8*) was identified and narrowed to an ~124-kb interval containing seven candidate genes on chromosome 8 in a near isogenic line (NIL) BC_3_F_2_ population.

## Introduction

Gray leaf spot (GLS), caused by different species of *Cercospora*, is a serious foliar disease of maize (*Zea mays L*.) globally, especially in the Americas and Africa (Ward et al., 1999; Crous and Braun, 2003; Crous et al., 2006). GLS was first identified in Asia in 2007 in Nepal (Manandhar et al., 2011). It also occurs in the spring production regions of maize in northeast China (Li and Mei, 2008) and has spread to the north, east, and southwest of China over the past 20 years. It has surpassed the occurrence of maize big spots in the Yunnan, Sichuan, and Hubei provinces of China and has become the most important leaf disease (Zhou et al., 2012). GLS is prevalent in production areas characterized by dew formation in the morning, followed by hot, humid afternoons, and relatively cool nights (Shrestha et al., 2019). GLS has become increasingly important and is currently seen as one of the most serious yield-limiting diseases of maize (Nutter Jr and Jenco, 1992; Ward and Nowell, 1998; Crous et al., 2006; Dhami et al., 2015). Previous studies have reported yield reductions of 260–320 kg/hm^2^ due to outbreaks of GLS (Engelsing et al., 2012).

Maize GLS is caused by various species of *Cercospora*, such as *Cercospora zeae-maydis* (*C. zeae-maydis*), *Cercospora zeina* (*C. zeina*), and *Cercospora sorghi* (*C. sorghi*) var. *maydis* (Wang et al., 1998). According to Wang et al. (1998) and Crous et al. (2006), the variation is rather low in populations of *C. zeae-maydis* and *C. zeina*, which are genetically and phenotypically distinct. In China, there are two *Cercospora* species causing GLS that are *C. zeae-maydis* and *C. zeina*. The former is found in northern China, such as, Heilongjiang, Jilin, Liaoning, Inner Mongolia, and Shandong provinces, and the latter was distributed in southwest China, such as Yunnan and Hubei provinces (Liu et al., 2013). Pathogen spores spread through the air and infect maize leaves, leaf sheaths, and bracts under high-temperature and high-humidity conditions (Zhang et al., 2012). Breeding GLS resistant cultivars is a prominent strategy being used to control this disease (Shrestha et al., 2019).

GLS resistance in maize is controlled by additive genetic effects, which is characteristic of quantitative trait inheritance (Juliatti et al., 2009; Du et al., 2020). In recent years, a number of quantitative trait loci (QTLs) studies on GLS resistance in maize have been conducted. As a result, a major QTL for GLS resistance has been identified on 10 different chromosomes in maize (Bubeck et al., 1993; Clements et al., 2000; Lehmensiek et al., 2001; Gordon et al., 2004; Pozar et al., 2009; Zwonitzer et al., 2010; Zhang et al., 2012). Results on the identification and stability of the QTLs, however, vary widely in different genetic backgrounds and environments. Shi et al. (2007) identified seven hotspot QTL and candidate genes on chromosomes 2, 3, and 8 of maize based on QTL meta-analysis. Wang et al. (2014) identified 11 consistent QTL intervals and three candidate genes for GLS resistance in maize through meta-analysis methods involving the homologous alignment of the maize genome with rice and *Arabidopsis thaliana* genomes.

Previous fine-mapping studies of QTL for GLS resistance have narrowed several QTL regions (Zhang et al., 2012, 2017; Xu et al., 2014; Benson et al., 2015; Lv et al., 2020; Sun et al., 2021). Zhang et al. (2012) identified two major QTLs, *qRgls1* and *qRgls2*, for GLS resistance on chromosomes 8 and 5, respectively, and fine mapped *qRgls1* to an interval of 1.4 Mb. In a subsequent study, Zhong (2018) narrowed the region of *qRgls1* to 120 kb on chromosome 8 in a 9 MB physical location of the B73 genome. This region was cloned by screening a bacterial artificial chromosome (BAC) library of the resistant parent. QTL-*qRgls2* was narrowed from an initial ~110-Mb region to an interval of ~1 Mb on chromosome 5, which harbored 15 predicted genes (Xu et al., 2014). Another QTL was finely mapped to an ~130-Kb region on chromosome 8 of a 152-Mb region of the B73 genome (Zhang et al., 2017). A previous study also narrowed QTL for GLS resistance to 5.2 and 6.5 Mb on chromosome 1 (Benson et al., 2015). Although the above studies have greatly advanced the identification of genetic markers for GLS resistance in maize, the discrepancies among different genetic backgrounds and environments underscore the need for further studies.

Molecular marker-assisted selection (MAS) represents an important strategy for breeding disease-resistant varieties. In the present study, QTL analyses and fine mapping in a Jiao 51 (J51, an elite inbred line) (Yang and Yang, 2007) near isogenic line (NIL) background was conducted and identified a quantitative trait locus for resistance to maize gray leaf spot (QTL*-qGLS8*) derived from T32 (an inbred line resistant to GLS derived from “Suwan”) (Wu et al., 2019). The present study provides new information on genetic markers for GLS resistance in maize that can be utilized for MAS breeding and provides a foundation for studying the functional role of candidate genes associated with GLS resistance in maize.

## Materials and Methods

### Plant Material and Field Experiments

The initial mapping population comprised 189 F_2 : 3_ families that originated from a cross between inbred T32 (a highly resistant genotype) and J51 (a highly susceptible genotype). F_2_ individuals were developed by planting F_1_ seeds in the winter of 2014 in Hainan, the southernmost province of China. Next, an F_2 : 3_ segregation population developed from selfing each F_2_ individual was planted in the spring of 2015 in Jiangdong (JD), Mengga (MG), and Mengwen (MW), in Yunnan, a southwest province of China. The F_2_ and the F_2 : 3_ families were used to conduct a QTL mapping analysis, which resulted in identifying an anti-disease position (named QTL-*qGLS*8) on chromosome 8 of maize (Yang, 2016).

**Figure 1:**
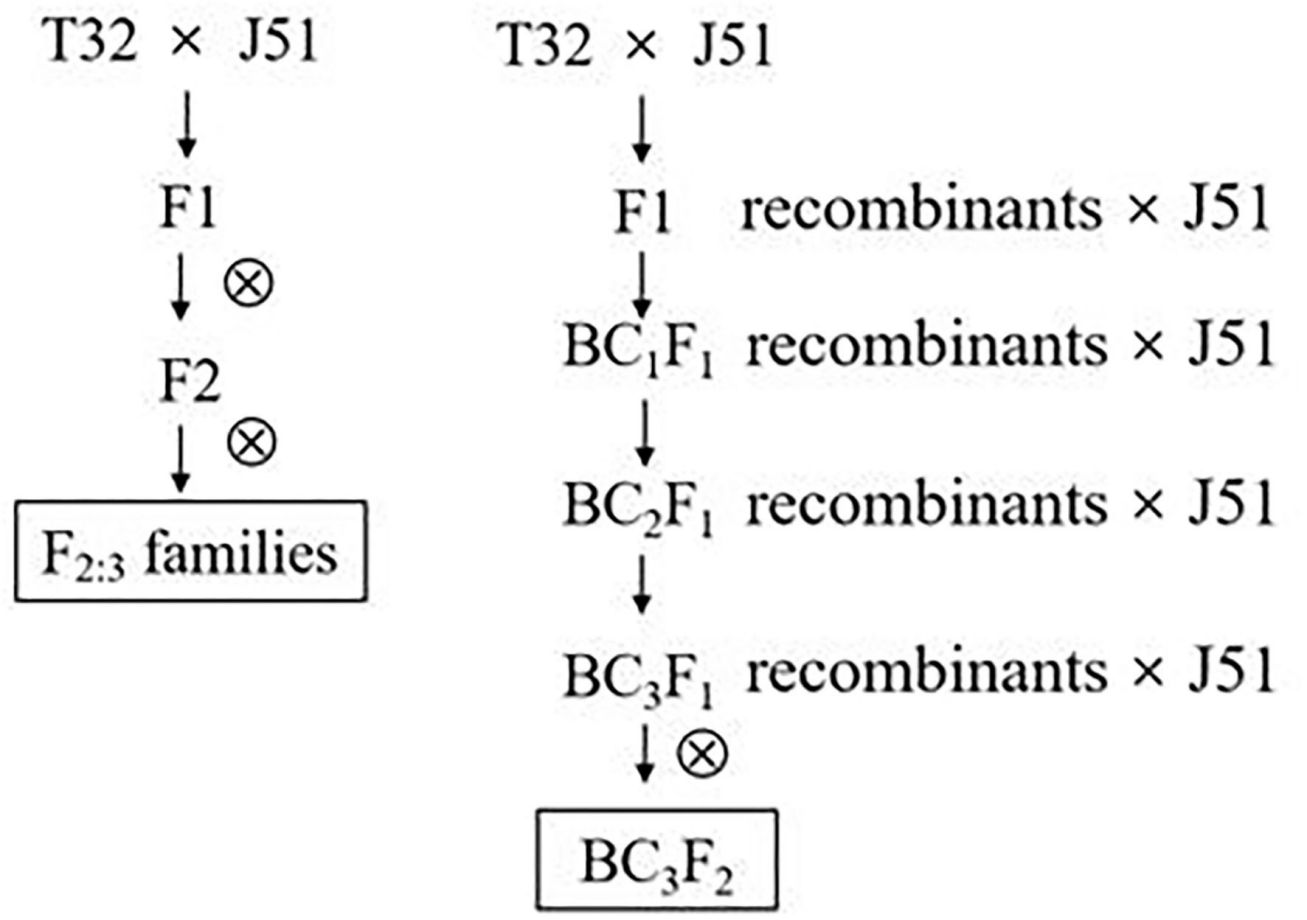
QTL mapping population used in the present study. QTL, quantitative trait loci.

Fine QTL mapping was performed by developing 20 BC_3_F_2_ lines, in which the recombination occurred within the region containing the target QTL. The original cross T32 (donor parent) × J51 (recurrent parent) was made in Hainan during the winter of 2013. Randomly selected F_1_ ears of the cross were pollinated by the recurrent parent J51 in the spring of 2014 to develop a backcross population (BC_1_F_1_). BC_1_F_1_ individuals were continuously backcrossed with the recurrent parent J51 using MAS until the generation of the BC_3_F_1_ population in the spring of 2015. Twenty individuals were selected from the BC_3_F_1_ population using simple sequence repeat (SSR) markers, denoted as D27, D128, D187, D221, D352, D355, D405, D428, D441, D493, D544, D550, D552, D600, D640, D647, D661, D707, D752, and D1123. The selected individuals formed a NIL population (named BC_3_F_2_-DQT) of 1468 plants, with BC_3_F_2_-D752 used to develop a NIL micro-population (named BC_3_F_2_-Micro) containing 180 plants. The NIL population was planted for phenotypic evaluation of GLS resistance in the spring of 2016 in JD, where a GLS epidemic was extreme (Figure 1).

### GLS Disease Evaluation

A total of 189 F_2 : 3_ families were evaluated for GLS resistance under natural conditions in two replicates at each of three locations, JD, MG, and MW. A scoring system with five levels (1, 3, 5, 7, and 9) was used to rank GLS resistance. A completely randomized block design with two replications and 20 plants in each plot was established in each of the three sites. All plants were self-pollinated, and 13 plants from the middle of each plot were sampled at 2- and 4-week post-pollination and ranked for GLS resistance (Table 1) (Zhang et al., 2012). The phenotypic evaluation of the BC_3_F_2_ population was the same as was used for the F_2 : 3_ population.

**Table 1:** Leaf spot disease classification criteria in China.

**Disease resistance level**	**Phenomenon of disease spot**	**Ratio of disease spot**
1 (HR)^a^	None or only a few disease spots	≤5%
3 (R)^b^	Small number of disease spots	6–10%
5 (MR)^c^	Increased disease spots	11–30%
7 (S)^d^	Numerous disease spots	31–70%
9 (HS)^e^	Leaves die, basically all of them are disease spots	≥71%

a *High resistance*,

b *resistance*,

c *medium resistance*,

d *diseased*,

e *highly diseased*.

### Statistical Analysis of the Phenotypic Data

Statistical analysis of the phenotypic data was performed using IBM SPSS Statistical 20.0 software. The variances for genotype, environment, and interaction between genotype and environment were estimated using Microsoft Office Excel based on a random model. The broad-sense heritability (*H*^2^) was calculated as follows: $H^{2}={\delta^{2}}_{G}/\left({\delta^{2}}_{G}+\frac{{\delta^{2}}_{GE}}{n}+{\delta^{2}}_{E}/nr\right)$, where $\delta_{\text{G}}^{2}$ represents the genetic variance, $\delta_{\text{GE}}^{2}$ represents the genotype and environment interaction, $\delta_{\text{E}}^{2}$ represents the error variance, and *n* and *r* are the numbers environments and replications, respectively (Knapp et al., 1985).

### Linkage Map Construction for the F_2_ Population

Genomic DNA was extracted from samples of young leaves of each plant using the cationic detergent cetyl-trimethyl ammonium bromide (CTAB) method (Chen and Ronald, 1999). The genotype of an individual was identified using a 10% non-denaturing polyacrylamide gel electrophoresis. A total of 286 polymorphic markers between T32 and J51 were used to develop the genetic map in JoinMap version 4 (Jacobs et al., 1995) based on publicly available SSR markers retrieved from the Maize Genetics and Genomics Database (http://www.maizegdb.org) (Portwood et al., 2019). The linkage map was constructed from 181 markers across 10 chromosomes. The recombination frequency between linked loci was transformed into the genetic distance (centimorgans, cM) through Kosambi's function.

### SSR Marker Development

For the QTL mapping of the BC_3_F_2_-micro population, QTL-*qGLS*8 was detected in a CI between SSR markers CSU329 and umc1034 with a physical distance of 1,102 kb according to the maize B73 whole-genome physical map (http://www.maizesequence.org/). Bacterial artificial chromosome sequences within the *qGLS*8 CI were downloaded from the website. These sequences were used to design new markers between the T32 and the J51 parent lines. The sequences were first scanned using SSRHunter1.3 software (Li and Wan, 2005) to identify SSRs. Primers were then designed using Primer Premier 5.0 software with the following criteria: primers should be ~20 nucleotides in length with a 40–60% guanine-cytosine content, there should be no consecutive tracts of a single nucleotide and no secondary structure. The primer sequences, mapping position, and the amplified length of the eight new markers developed in this study are listed in Table 2.

**Table 2:** Newly developed SSR markers in the QTL-*qGLS*8 target segment.

**Marker**	**Physical coordinate (bp)**	**Motif length**	**Size (bp)**	**Forward primer**	**Reverse primer**
ym20	21237068	TTTC (5)	141	GGACCCAAAAGGCATCTTCAG	GCAGCACTGCTCGAATAAAAAC
ym21	21238107	TAGA (6)	181	GTTTTACCTTGCTGCACGTCAT	CGCAGGTTTCTCTGCTTACCAT
ym31	21509708	CAG (5)	166	AAGCAGACGGGGAAACAAGAAT	GTTCCTCGAGCTTCTCGTTGAT
ym37	21605276	TC (5)	159	TGTAGATGACCAAACCTCTTTGC	TTGGAGTTGAAGAACAGGTCAGT
ym51	21361115	GGC (6)	186	GGTGGATTTGTTTTGGGGTTCT	CAGGAACACCAAAGCTGGAAAT
ym71	21501955	CG (5)	162	GGGCGCATCAAGTTAACACAAC	TGACCTGACAACTCCTCCCAAT
ym73	21509601	TAC (6)	272	CTCCGTTCACTTGCTTGTTGTT	TTCCTCGAGCTTCTCGTTGATG
ym75	21509708	TAC (6)	160	AAGCAGACGGGGAAACAAGAAT	CGAGCTTCTCGTTGATGAGCTT

*Position of the reference Zea mays B73_RefGen_v4*.

*SSR, simple sequence repeat; QTL-qGLS8, quantitative trait locus for resistance to maize gray leaf spot*.

### QTL Analysis

Inclusive composite interval mapping was performed using QTL IciMapping software (Li et al., 2007) to identify the QTL and estimate their effect. Parameters for forward regression analysis were set with window size and walking speed of 10 and 1 cM, respectively. The significance threshold for QTL detection included 1,000 random permutations of the phenotypic data at the 5% level. The gene action mode of each significant QTL was estimated in accordance with the rate (D/A) of additive (A) and dominant (D) effects and classified as additive (*A* = 0–0.20), partial dominant (PD = 0.21–0.80), dominant (*D* = 0.81–1.20), and over-dominant (OD > 1.20) (Edwards et al., 1987; Tuberosa et al., 1998). QTLs were named using a standardized approach, for example, in JD*GLS4a*, “JD” represents the environment (site) in which the QTL was identified (JD, MG, and MW are abbreviations for the Jiangdong, Mengga, and Mengwen planting sites, respectively); “GLS” represents the name of the trait; the number “4” represents the serial number of the chromosome on which the QTL is located; and “a” represents the serial alpha code of the detected QTL.

A total of 1,468 BC_3_F_2_ plants were genotyped using 10 SSR markers to construct a small-scale linkage map. Eight of the SSR markers were newly developed. The small-scale genetic mapping of the *qGLS*8 genes was performed using JoinMap version 4 software to combine the genotypic data of molecular markers with QTL-*qGLS*8 genes.

## Results

### Phenotypic Variation of GLS Resistance in the F_2 : 3_ Families

The *t*-tests of the differences in GLS resistance in the two parents within a site were highly significant (*P* < 0.01; Table 3). GLS resistance in the T32 parent was significantly higher than it was in the J51 parent. The scores for GLS resistance exhibited continuous and approximately normal distributions across the three sites with low skewness and kurtosis, indicating that GLS resistance is a typical quantitative trait (Table 3; Supplementary Figure 1). A transgressive segregation of the resistance score was observed in the populations across the three sites. In addition, variance analysis indicated that the effect of genotype (F_2 : 3_ families), environment, and a genotype/environment interaction on GLS resistance was highly significant (*P* < 0.01), and that heritability was >80% (Table 4).

**Table 3:** Phenotypic ranking of GLS resistance in F_2 : 3_ families and their parents grown in three environments (sites).

**Site**	**Parents**		**F**_**2:3**_					
	**T32**	**J51**	**Mean**	**Range**	**SD^a^**	**Skewness**	**Kurtosis**	**W^b^**
JD	3^**^	9	7.6008	5–9	0.9778	−0.445	−0.443	0.962
MG	3^**^	9	6.4241	3.5–9	1.0407	0.158	−0.048	0.991
MW	3^**^	9	6.9750	4.25–9	0.9668	−0.084	−0.451	0.991

a *Standard deviation of phenotypic data*,

b *Shapiro–Wilk statistic for the W test of normality*,

** *significance with P < 0.01*.

*GLS, Gray leaf spot*.

**Table 4:** ANOVA of GLS resistance in F_2 : 3_ families grown in three environments (sites).

**Source**	**DF^a^**	**Stdev square**	**Mean square**	***F* value**	***P* value**
Inter-familial	188	862.829	3.967	5.780	0.0001
Location	2	241.670	120.835	152.169	0.0001
Family × environment	371	291.019	0.784	0.988	0.001
Error	377	299.369	0.794		

*GLS, Gray leaf spot*.

a *Degree of freedom*.

### Linkage Map of the F_2_ Population

**Figure 2:**
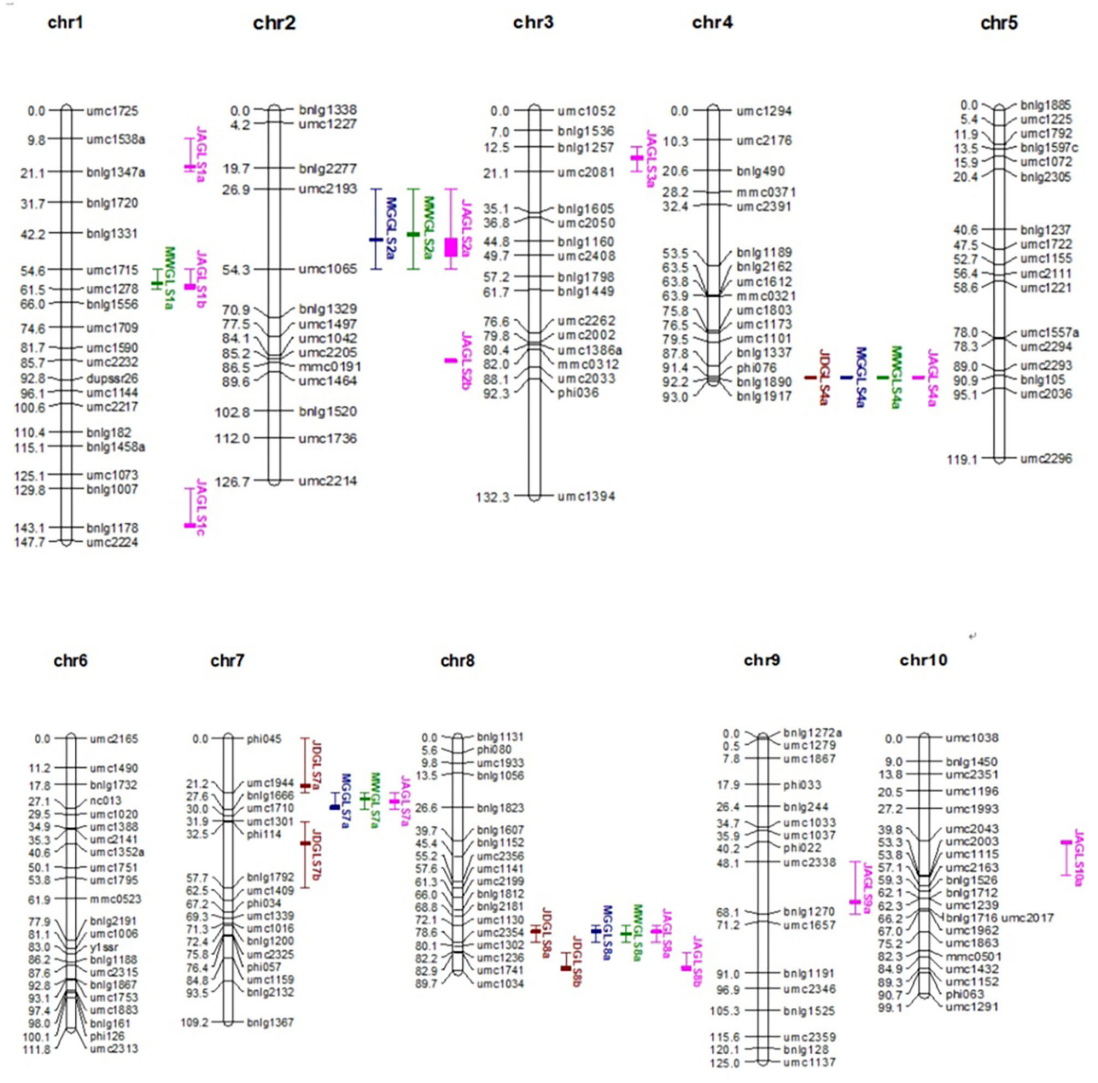
Distribution of GLS resistance QTL on the linkage map of the F_2 : 3_ population. QTL, quantitative trait loci; GLS, gray leaf spot.

The length of the QTL map constructed using the 181 polymorphic SSR markers (Figure 2) was 1153.497 cM, and the average distance between markers was 6.37 cM. The average number of markers per chromosome was 18.1, ranging from 16 on chromosome 2 to 22 on chromosome 6. A few differences in marker order on a chromosome were compared to the IBM 2008 Neighbors Frame 6 (Supplementary Figure 2). These variations in distance and order may be due to the different materials used in the population.

### Single Environment Analysis of the GLS Resistance Main-Effect QTL in the F_2 : 3_ Families

Fourteen QTLs for GLS resistance were detected in F_2 : 3_ families, which comprised five QTLs at JD, four QTLs at MG, and five QTLs at MW (Table 5). Two of the loci were detected in different environments. JD*GLS4a*, MG*GLS4a*, and MW*GLS4a* were located in a common genetic region on chromosome 4 within the Phi076–bnlg1890 marker interval (bin4.11) and explained 5.86, 6.75, and 6.20% of the phenotypic variation in GLS resistance at the JD, MG, and MW sites, respectively. Additive effects of three QTLs on increased GLS resistance were contributed by T32, whose effect values were −0.34, −0.32, and −0.36, respectively. JD*GLS8a*, MG*GLS8a*, and MW*GLS8a* were also located in a common genetic region on chromosome 8 in the umc1130–umc2354 marker interval (bin8.03) and explained 7.89, 14.71, and 11.51% of the phenotypic variation in GLS resistance at the JD, MG, and MW sites, respectively. Additive effects of three QTL on increased GLS resistance were also contributed by T32, whose effect values were −0.22, −0.61, and −0.46 respectively. Compared to *qGLS8*, the effect value of *qGLS4* was lower. Therefore, *qGLS8* was used as the main locus for further study. The additive effects of the T32 allele in 86% of the QTL increased the GLS resistance level by 0.22–0.61, compared to the additive effect of the J51 allele. The modes of gene action were A, PD, and OD.

**Table 5:** QTL associated with GLS resistance in a single environment analysis of the F_2 : 3_ population.

**Site**	**QTL name**	**Bins**	**Interval markers**	**Position**	**LOD**	***R*^**2**^ (%)^a^**	**Additive effect^b^**	**Dominance effect**	**Gene action**
JD	JD*GLS4a*	4.11	Phi076–bnlg1890	92	3.45	5.86	−0.34	−0.05	PD
JD	JD*GLS7a*	7.05–7.04	phi045–umc1944	19	2.86	4.60	−0.26	0.22	D
JD	JD*GLS7b*	7.03–7.02	phi114–bnlg1792	41	2.58	5.46	−0.28	−0.21	PD
JD	JD*GLS8a*	8.03	umc1130–umc2354	75	2.53	7.89	−0.22	0.43	OD
JD	JD*GLS8b*	8.03–8.02	umc1741–umc1034	89	3.56	6.18	−0.38	−0.01	D
MG	MG*GLS2a*	2.02–2.06	umc2193–umc1065	45	3.52	8.98	0.35	−0.35	PD
MG	MG*GLS4a*	4.11	Phi076–bnlg1890	92	4.37	6.75	−0.32	0.03	PD
MG	MG*GLS7a*	7.04	umc1944–bnlg1666	27	3.59	7.86	−0.41	0.21	PD
MG	MG*GLS8a*	8.03	umc1130–umc2354	75	5.84	14.71	−0.61	−0.06	A
MW	MW*GLS1a*	1.09–1.07	umc1715–umc1278	60	2.71	4.25	−0.28	−0.09	PD
MW	MW*GLS2a*	2.02–2.06	umc2193–umc1065	43	4.43	8.55	0.31	−0.33	D
MW	MW*GLS4a*	4.11	phi076–bnlg1890	92	4.21	6.20	−0.36	0.05	A
MW	MW*GLS7a*	7.04	umc1944–bnlg1666	24	3.33	5.13	−0.32	−0.01	A
MW	MW*GLS8a*	8.03	umc1130–umc2354	76	7.22	11.51	−0.46	0.21	PD

a *Phenotypic variance explained (%), i.e., percentage of phenotypic variance explained by the QTL*,

b *A, additive effect of the QTL, negative values indicate that the alleles for increasing the GLS resistance are contributed by T32, positive values indicate that the alleles for increasing the GLS resistance are contributed by another parent J51*.

*GLS, Gray leaf spot; QTL, quantitative trait locus*.

### Joint Analysis of QTL Detection in the F_2 : 3_ Families Across Three Environments

Twelve QTLs were detected for GLS resistance in the joint analysis combining data from all three sites (Table 6). The two common genetic regions detected in the single environment (site) analysis were also detected in the joint analysis within the same marker intervals with the relatively higher log of odd (LOD) values of 8.25 and 10.11. The two QTLs detected in only one environment and the one QTL detected only in two environments (sites) in the single environment analysis were also detected in the joint analysis within the same marker intervals. One common genetic regions detected in the MG and MW sites in the single environment analysis was also detected in the joint analysis, located near the genetic region of QTL-JD*GLS7a*, which was detected only at the JD site with the same marker (umc1944). Some QTLs detected in the joint analysis across all three environments (sites) were not detected in the single environment analysis. These QTLs probably have a minor effect and are detected when the analysis includes a greater number of environments.

**Table 6:** QTL associated with GLS resistance in a joint analysis across all three environments in the F_2 : 3_ population.

**Chr**	**QTL name**	**Bins**	**Interval markers**	**Position**	**LOD score(A)^a^**	**LOD score (A by E)^b^**	**Est A**	**Est D**	**Gene action**
1	JA*GLS*1a	1.11–1.1	umc1538a–bnlg1347a	20	2.17	0.85	0.01	0.21	OD
1	JA*GLS*1b	1.09–1.07	umc1715–umc1278	61	5.33	0.53	−0.24	−0.09	PD
1	JA*GLS*1c	1.02	bnlg1007–bnlg1178	143	5.82	0.88	0.24	0.08	PD
2	JA*GLS*2a	2.02–2.06	umc2193–umc1065	51	3.82	1.61	0.18	−0.10	PD
2	JA*GLS*2b	2.07	umc2205–mmc0191	86	1.78	1.66	0.04	−0.18	OD
3	JA*GLS*3a	3.09–3.08	bnlg1257–umc2081	17	2.50	0.99	−0.10	0.17	OD
4	JA*GLS*4a	4.11	phi076–bnlg1890	92	8.25	0.62	−0.32	0.02	A
7	JA*GLS*7a	7.04	umc1944–bnlg1666	25	7.62	1.18	−0.29	0.03	A
8	JA*GLS*8a	8.03	umc1130–umc2354	75	10.11	5.15	−0.32	0.17	PD
8	JA*GLS*8b	8.03–8.02	umc1741–umc1034	89	3.00	1.66	−0.19	0.08	PD
9	JA*GLS*9a	9.03–9.04	umc2338–bnlg1270	64	3.30	0.36	−0.17	−0.08	PD
10	JA*GLS*10a	10.04	umc2043–umc2003	40	2.74	0.28	0.11	−0.20	OD

a *LOD (A), LOD score for additive and dominance effects*,

b *LOD (A by E), LOD score for additive and dominance by environment effects, Additive effect represents the average of the three additive effects of the QTL in each of the three environments, Dominance effect represents the average of the three dominance effects of the QTL in each of the three environments*.

*GLS, Gray leaf spot; QTL, quantitative trait locus*.

### Fine Mapping of *qGLS*8

Randomly selected F_1_ ears of the original T32 (donor parent) × J51 (recurrent parent) cross were pollinated by the recurrent parent J51 to develop a backcross population (BC_1_F_1_). BC_1_F_1_ individuals were continuously backcrossed with the recurrent parent J51 using MAS until the BC_3_F_1_ generation was obtained. Twenty individuals were selected from the BC_3_F_1_ population using SSR markers (designated as umc1130 and umc2354). The BC_3_F_2_ population, developed from selfing each BC_3_F_1_ individual, was used to identify recombinants for fine mapping. In 20 individuals, the T32 chromosomal segment harboring *qGLS*8 was present in the genetic background of J51. A total of 180 BC_3_F_2_-Micro individuals were genotyped using 12 SSR markers located in the target region and evaluated for GLS resistance. Fine mapping confirmed a QTL peak between markers CSU329 and umc1034 with an LOD score of 4.55 and an *R*^2^ of 11.11% (Table 7). This peak differed from the results of the original F_2 : 3_ mapping (Tables 5, 6), which may have been due to the increased SSR marker density used in the fine mapping and the elimination of interference from the genetic background.

**Table 7:** QTL analysis of *qGLS8* in different populations.

**Population**	**No of plants**	**Position**	**Left marker**	**Right marker**	**LOD**	***R*^**2**^ (%)**	**Additive effect**	**Dominance effect**	**Gene action**
BC_3_F_2_-Micro	180	3	CSU329	umc1034	4.55	11.11	−0.36	−0.47	OD^a^
BC_3_F_2_-Micro	180	2	ym20	ym31	4.34	10.51	−0.35	−0.34	D^b^
BC_3_F_2_-DQT	1,468	0	ym20	ym51	58.61	17.46	−0.79	−0.45	PD^c^

a *Over dominant*,

b *dominant*,

c *partially dominant*.

*QTL, quantitative trait locus*.

The phenotypic means of BC_3_F_2_-Micro individuals were compared among the three genotypic classes defined by the allele constitution of markers in the CSU329–umc1034 interval to confirm the gene action of the QTL-*qGLS*8 identified in this study. The difference in the level of GLS resistance between plants having a homozygous T32 introgression in the target region and those having a homozygous introgression of J51 DNA was 0.61 (a difference of 8.24%). These results confirmed the identification of the primary QTL in the F_2 : 3_ population and demonstrated that the transgressive segregant could be identified in subsequent generations. The mean value of GLS resistance in the homozygous T32 class (7.40) was not significantly different from that of the heterozygous class (7.37); however, both were significantly different from that of the homozygous J51 class (8.01, *P* < 0.01). These results indicate that the T32 allele was completely dominant over the J51 allele. This analysis implied that the homozygous T32 class and the heterozygotes could be combined for use in the phenotypic analysis during fine mapping, which provided a larger sample size and greater statistical power.

Four newly developed polymorphic SSR markers were added to the existing SSR markers to genotype BC_3_F_2_-Micro individuals to precisely define the location of the critical breakpoints. A high-resolution genetic linkage map was constructed in the target region. QTL analysis confirmed the highly significant peak between markers ym20 and ym31 with an LOD score of 4.34 and an *R*^2^ value of 10.51% (Table 7).

**Figure 3:**
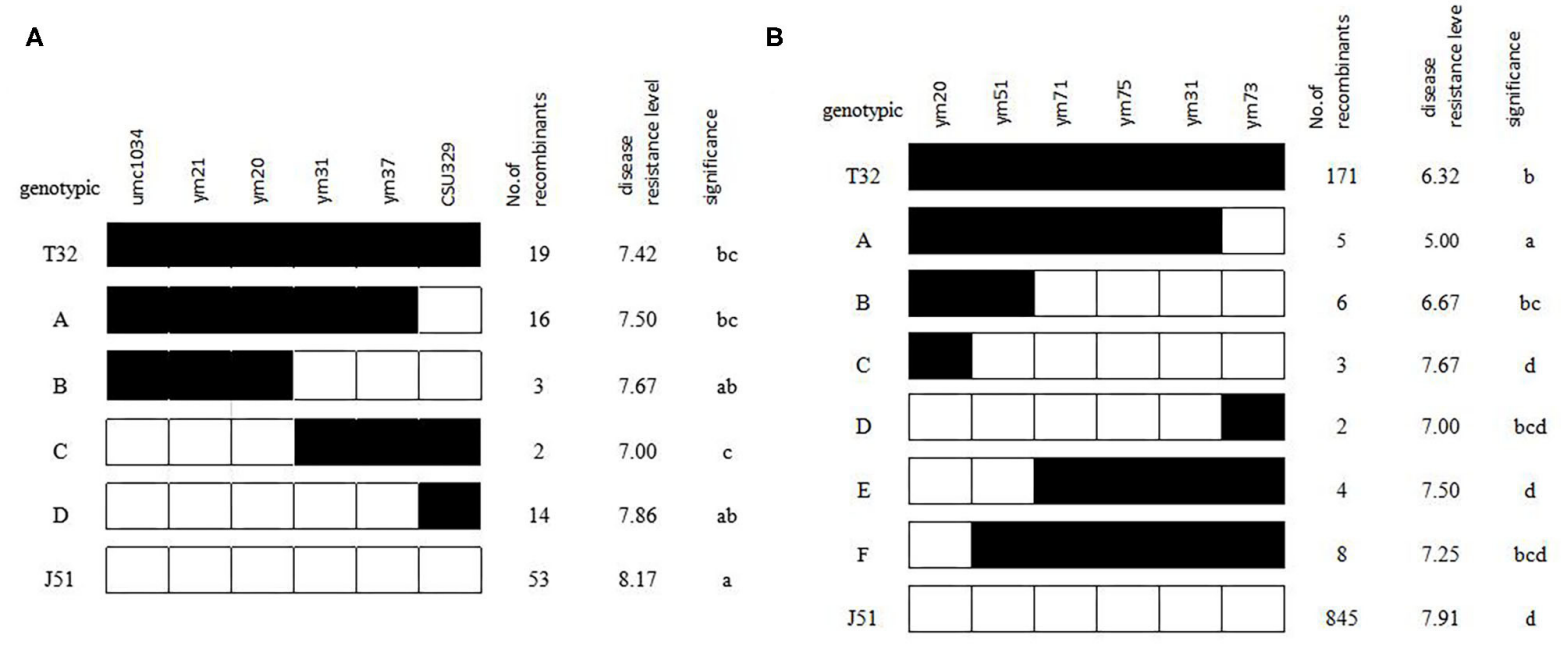
Fine mapping of *qGLS8* in the BC_3_F_2_-Micro **(A)** and BC_3_F_2_-DQT **(B)** population.

In addition to the QTL analysis, an extreme sampling strategy was used to clarify on which side of ym20–ym31 the *qGLS8* locus was located. A total of 180 BC_3_F_2_-Micro individuals were compared genotypically using six of the markers in the target region. Based on these data, 35 recombinants were identified (Figure 3A). The mean phenotypic performance of individuals with and without the T32 introgression was compared, and results indicated that groups B and C were significantly different (*P* < 0.05). Thus, the ym20–ym31 interval represents a critical region of recombination, with the critical breakpoint falling between ym20 and ym31. This finding further supported the results of the QTL linkage analysis.

A total of 1,468 BC_3_F_2_ plants were genotyped for the fine mapping of *qGLS8* and included the use of four new markers developed to further subdivide the target interval. The QTL analysis confirmed the presence of an extremely significant peak between markers ym20 and ym51 with LOD and *R*^2^ values of 58.61 and 17.46%, respectively (Table 7). A total of 28 recombinants were identified and grouped into six genotypic classes consisting of 5, 6, 3, 2, 4, and 8 individuals. When the phenotypic means for each class were compared with the T32 and J51 parents (Figure 3B), the most informative comparison was between classes B and C, where the phenotypes (between T32 and J51) were significantly different (*P* < 0.05). Based on these results, the location of *qGLS*8 was concluded to be in the recombination area between ym20 and ym51. This conclusion was further supported by the comparison between classes B and E. Consequently, the location of *qGLS*8 was narrowed down to a 124 kb fragment defined by ym20 and ym51 markers on chromosome 8.

**Figure 4:**
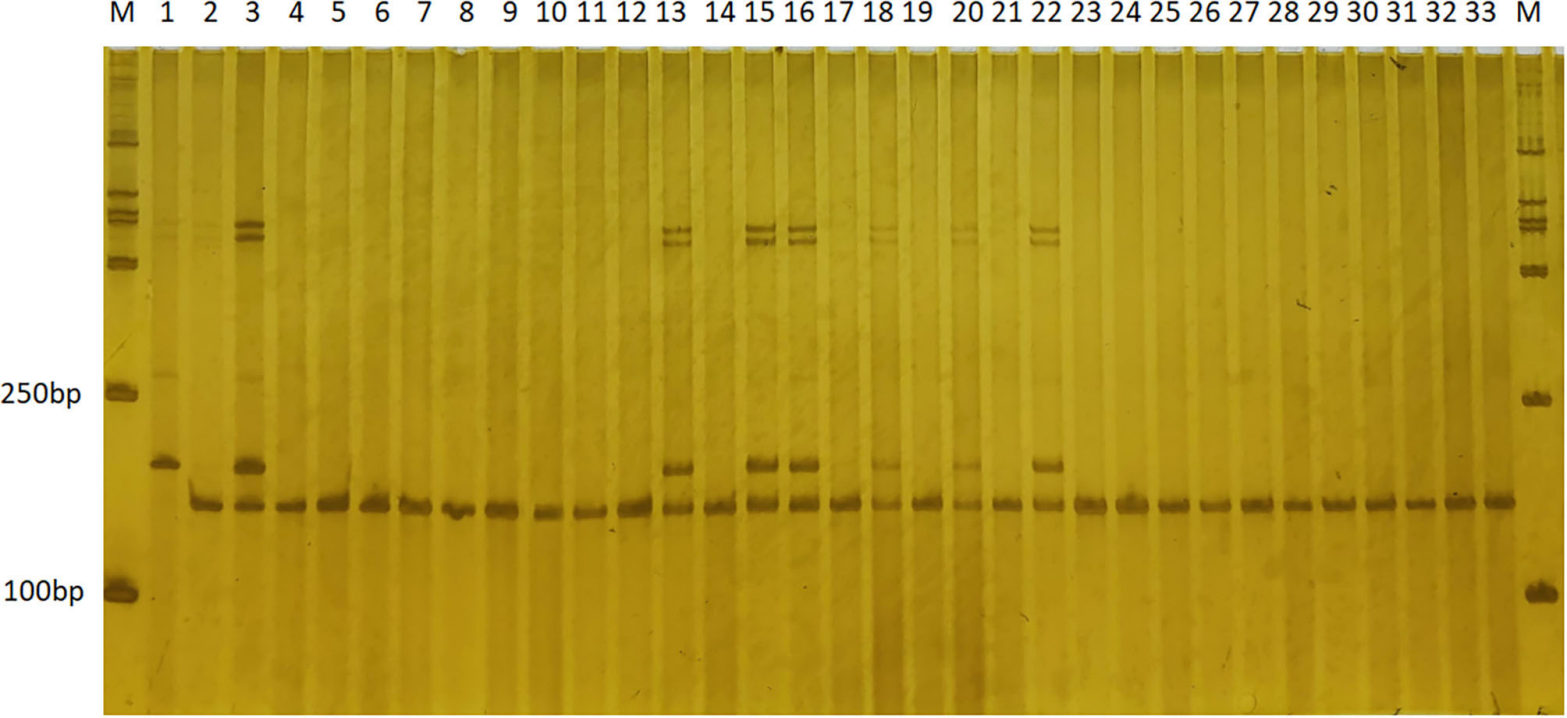
The application of SSR marker ym51 in genotyping the BC_5_F_1_ population by electrophoresis on a PAGE gel. Polymorphic bands were observed in the two parental lines, “T32” (*lane 1*), “J51” (*lane 2*), and both representative bands in the “F1” (*lane 3*). Variations on the banding pattern were observed among the BC_5_F_1_ individuals (*lanes 4–33*). *Lanes 4–33* are randomly selected BC_5_F_1_ individuals that are either homozygous (one band having the same size as that of “J51”) or heterozygous (two bands corresponding to “T32” and “J51,” respectively). SSR, simple sequence repeat.

The eight newly developed markers were authentically mapped on bin 8.03 with two of the markers (ym20 and ym51) within and six of the markers located outside of the resistance *qGLS*8 region. The newly developed markers will greatly facilitate both map-based cloning and MAS breeding for GLS resistance in maize (Figure 4). The 124-kb region harbors seven genes based on the B73 Reference-Gramene_v4 (Tello-Ruiz et al., 2018) and the Maize GDB, including five genes and two lincRNAs (Table 8).

**Table 8:** Information on candidate genes present within the ~124-kb region of QTL-*qGLS8*.

**Gene id**	**Start–end^a^**	**Gene type**	**Also known as**	**Gene description^b^**
Zm00001d008808	21238766…21240538	Protein coding	GRMZM2G049695	MYB-related transcription factor 24
Zm00001d008809	21249236…21251762	Protein coding	GRMZM2G402211	Pyrophosphate–fructose 6-phosphate 1-phosphotransferase subunit alpha 2
Zm00001d008810	21254507…21266662	Protein coding	GRMZM2G102490	Unknown
Zm00001d008000	21264425…21267255	lincRNA	-	Unknown
Zm00001d008811	21268689…21273710	Protein coding	-	Unknown
Zm00001d008001	21268760…21273571	lincRNA	-	Unknown
Zm00001d008812	21359783…21360919	Protein coding	GRMZM2G173124	C_3_H–transcription factor 347

a *Position of reference Maize B73 Reference–Gramene_v4*,

b *Information derived from the Gramene database*.

*QTL-qGLS8, quantitative trait locus for resistance to maize gray leaf spot*.

## Discussion

Maize, one of the primary cereal crops produced for both human and animal consumption, suffers from numerous biotic and abiotic stresses, which may result in considerable reductions in yield (Dhami et al., 2015). GLS is a major threat to maize production worldwide (Lv et al., 2020). GLS resistance in maize is a typical quantitative trait (Zhang et al., 2017) and the identification of major QTL and candidate genes is crucial to the use of marker-assisted breeding for GLS resistance.

Previous studies have reported more than 100 QTLs for GLS resistance distributed on all 10 chromosomes of maize (Du et al., 2020). The QTL on chromosome 8, *QTL8*, is defined by markers npi590A–umc93 (4.7 cM) and umc48–umc30 (6.9 cM) in F_2_ and F_2 : 3_ populations and explains 7.7–11.0% of the phenotypic variance in GLS resistance (Maroof et al., 1996). Shi et al. (2007) detected a consensus QTL (446.14 cM) in Bin 8.06 using a meta-analysis of five previous studies. In addition, two of the loci of *QTL8* are defined by markers PZA01470.1 (Bin 8.03) and PZA03651.1 (Bin 8.06) using nested association mapping, and six candidate genes have been identified encoding the Rust resistance protein rp3-1, Heat shock factor-binding protein 1, GapC2, T cytoplasm male sterility restorer factor 2, Chloroplast phytoene synthase, and B transcriptional activator (Benson et al., 2015). The fine mapping of *qGLS8* has indicated that *qGLS8* is defined by the markers ctg358-32 and ctg358-01, located in an ~130-kb region in Bin 8.06, and contains five genes encoding the Receptor-like protein kinase 2, Phosphoethanolamine N-methyltransferase 3, Receptor-like protein kinase 2, ABC transporter, and an uncharacterized protein (Zhang et al., 2017).

In the present study, two populations of maize were developed to identify major QTL for GLS resistance in the F_2 : 3_ population derived from the T32 parent and to conduct fine mapping in a BC_3_F_2_ population with a J51 NIL background. The T32 and J51 parents may provide novel, major resistance genes. In our study, *qGLS8* was detected as the only overlapping QTL, defined by the markers umc1130 and umc2354 (Bin 8.03), in different environments (sites) and explains up to 12.98% of the phenotypic variation for GLS resistance in the F_2 : 3_ population. In the BC_3_F_2_ population, *qGLS8*, defined by the ym20 and ym51 (Bin 8.03) markers, explains up to 17.46% of the phenotypic variation in GLS resistance. The genetic effect of *qGLS8* is significant and stable in the F_2 : 3_ and BC_3_F_2_ populations across multiple environments, suggesting that it is a major QTL for GLS resistance in maize.

In the present study, QTL-*qGLS8* was narrowed down to a 124-kb fragment defined by markers ym20 and ym51 with a physical location of 21.23 Mb and 21.36 Mbp on chromosome 8, respectively, according to the B73 reference genome. The QTL detected in our study is different from the QTL previously reported by Zhang et al. (2012), with a physical location on chromosome 8 from 8.61 Mb to 10.07 Mbp, according to the B73 reference genome. Moreover, the QTL detected in the physical location from 23.77 Mbp to 101.18 Mbp by Benson et al. (2015) does not harbor the QTL-*qGLS8* identified in the present study. Notably, the interval of the QTL identified in the present study does not overlap the map position for GSL resistance reported in other studies, indicating that it represents the discovery of a new major locus for GLS resistance, potentially resulting from the introgression of T32. The major QTL-*qGLS8* in bin 8.03 represents a good choice for resistance gene cloning and for use in marker-assisted selection in breeding programs focusing on GSL resistance.

Seven candidate genes were identified in the QTL-qGLS8 region, including two uncharacterized lincRNAs and five genes, according to the B73 Reference-Gramene_v4. *Zm00001d008808*, encoding the MYB-related transcription factor 24 is significantly upregulated in response to drought (Zenda et al., 2019) and long-day conditions (Ku et al., 2016), suggesting that it may be a stress-responsive gene. The identified MYB-related transcription factor has also been shown to function in regulating the circadian clock in Arabidopsis (Nguyen and Lee, 2016) and promoting flowering in rice (Zhang et al., 2016), which corresponds to previous research that found a strong correlation between flowering time and GLS resistance (Zwonitzer et al., 2010). *Zm00001d008812*, encoding the C3H-transcription factor 347, has been reported to be involved in cell wall biogenesis as a differentially expressed gene in elongating vs. non-elongating maize internodes (Bosch et al., 2011). C3H-transcription factors also contribute to salt stress tolerance in many plants (Jiang et al., 2017; Liu et al., 2020), which may reflect its role in the adaptation to stress response. The potential function of the other candidate genes, however, is unclear. The function of candidate lincRNAs in maize is also unclear; however, lincRNAs have been shown to play an important role in plant development and response to stress in *Arabidopsis* (Li et al., 2016). The function and the regulatory mechanisms of candidate genes and lincRNAs in GLS resistance require further study. Our future studies will focus on the functional role of the candidate genes *Zm00001d008808* and *Zm00001d008812* and involve gene cloning, expression analysis, overexpression studies, and other molecular approaches. The fine mapping of the newly identified QTL QTL-*qGSL8* provides further insight into GLS resistance in maize and could play an important role in developing maize varieties with increased resistance to GLS.

## Data Availability Statement

The original contributions presented in the study are included in the article/Supplementary Material, further inquiries can be directed to the corresponding author/s.

## Author Contributions

HQ and GB contributed conception and designed the study. HQ, CL, and WY performed the field experiments. CL, WY, and KT assayed phenotype under controlled growth conditions and assembled and analyzed the data. HQ and CL wrote the manuscript. All authors contributed to manuscript revision.

## Funding

This work was supported by the National Natural Science Foundation of China (31460384) and (32060488).

## Conflict of Interest

The authors declare that the research was conducted in the absence of any commercial or financial relationships that could be construed as a potential conflict of interest.

## Publisher's Note

All claims expressed in this article are solely those of the authors and do not necessarily represent those of their affiliated organizations, or those of the publisher, the editors and the reviewers. Any product that may be evaluated in this article, or claim that may be made by its manufacturer, is not guaranteed or endorsed by the publisher.

## Abbreviations

GLS: Gray leaf spot
QTLs: Quantitative trait loci
SSR: Simple sequence repeat
NIL: Near isogenic line
MAS: Marker-assisted selection
JD: Jiangdong
MG: Mengga
MW: Mengwen
cM: Centimorgans
QTL-qGLS8: An anti-disease position on chromosome 8 in maize.
