# Supplementary material for: Fine Mapping of a New Major QTL-qGLS8 for Gray Leaf Spot Resistance in Maize
Source: Front Plant Sci. 2021 Sep 17;12:743869. doi: 10.3389/fpls.2021.743869 (PMC8484643; doi:10.3389/fpls.2021.743869)
Supplement: Supplementary file 1 [file Data_Sheet_1.docx]

**Supplemental Figure.1** Frequency distribution of the grade of GLS resistance in the F_2:3_ population


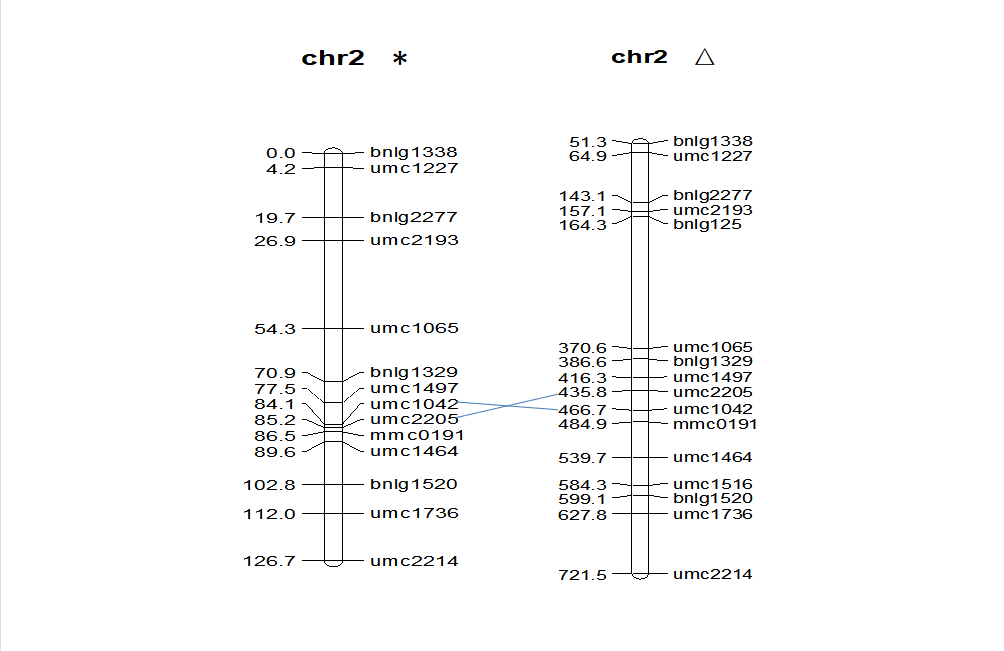

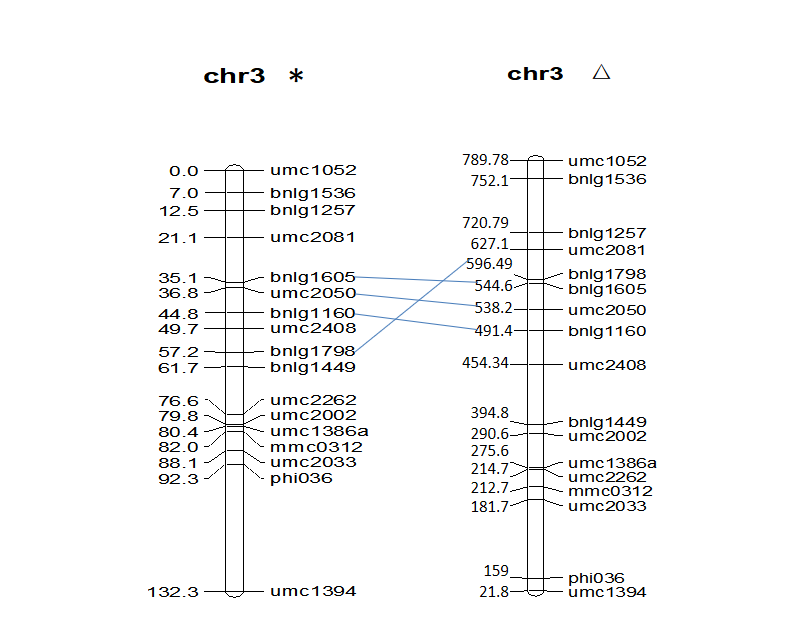

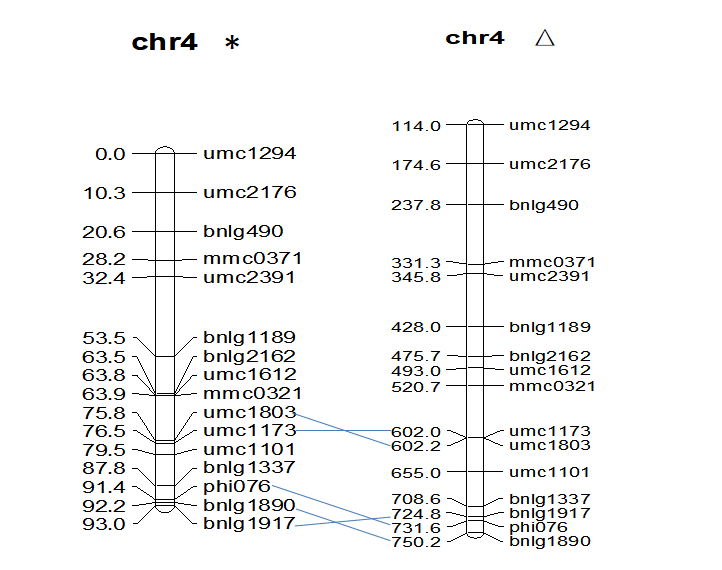

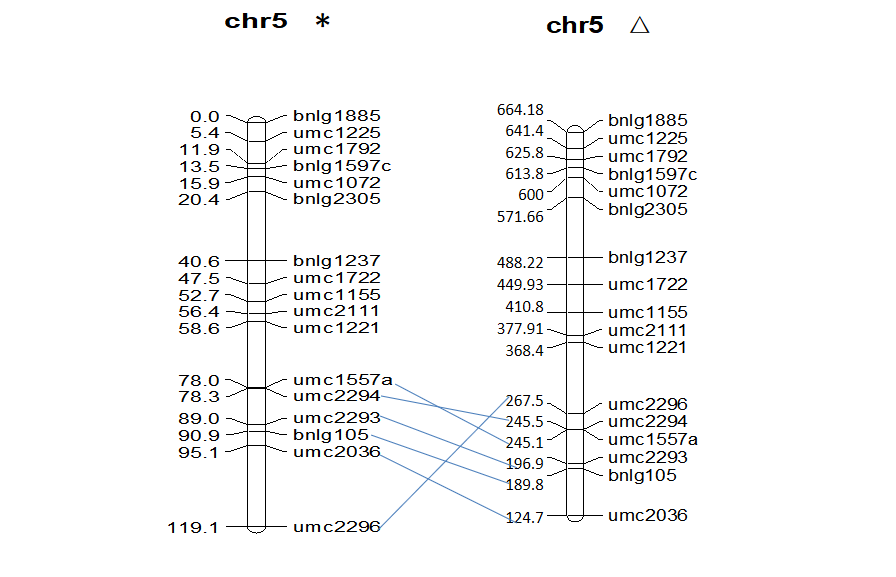


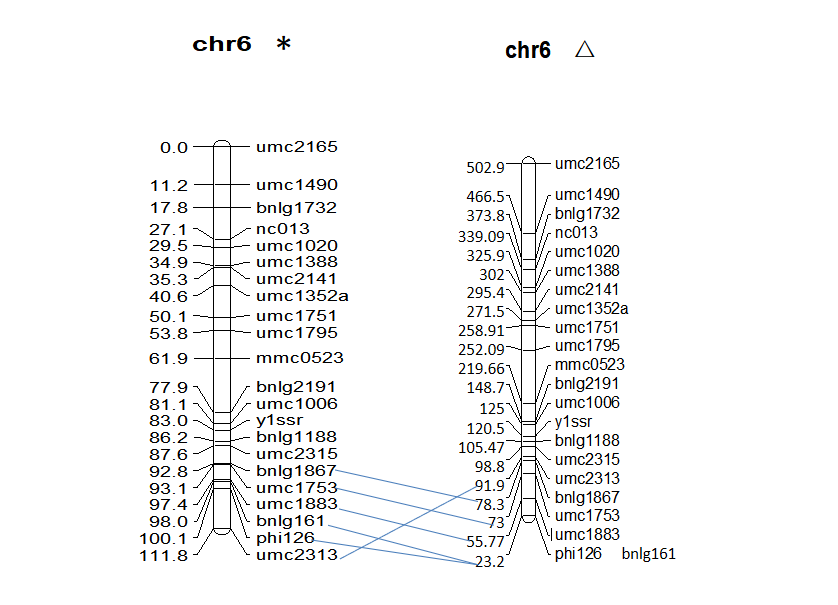

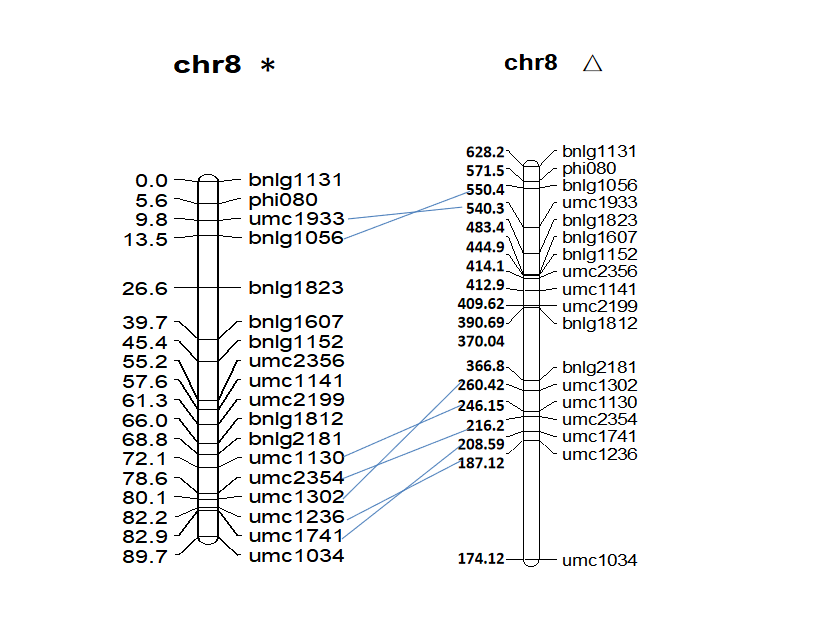


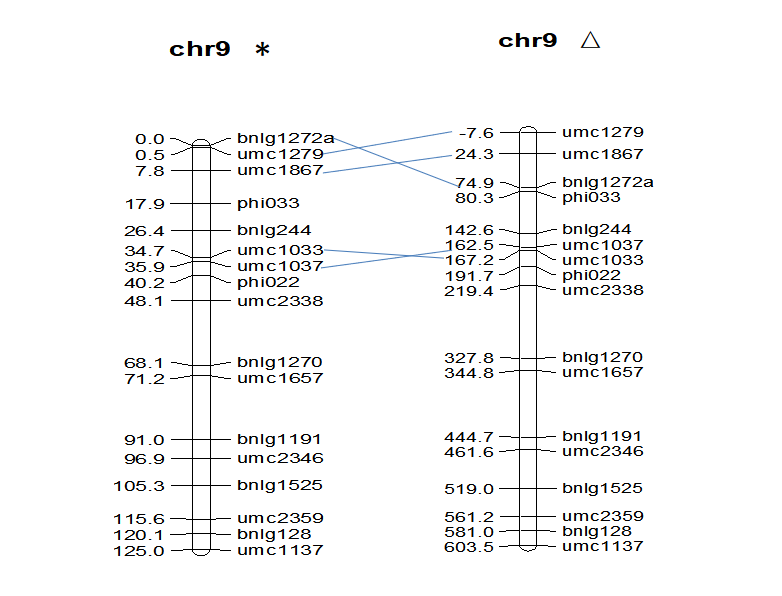

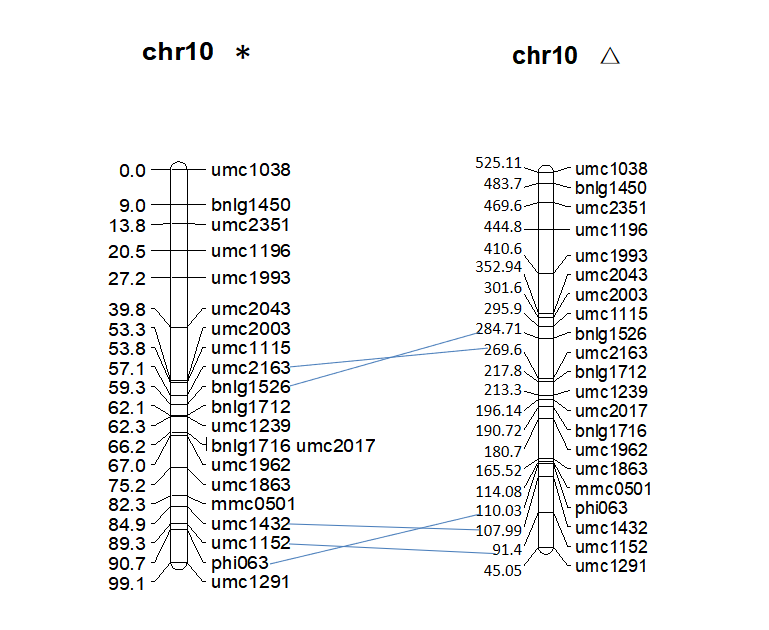


**SupplementaRY Figure 2.** Differences in the order of the SSR markers in the linkage map of the F_2_(T32 X J51) population compared to the IBM 2008 Neighbors Frame 6. * Represents the linkage map of the F_2_(T32 X J51) population, △ Represents the IBM 2008 Neighbors Frame 6 results.
